# Immune checkpoint regulator PD-L1 expression on tumor cells by contacting CD11b positive bone marrow derived stromal cells

**DOI:** 10.1186/s12964-015-0093-y

**Published:** 2015-02-27

**Authors:** Hyangsoon Noh, Jiemiao Hu, Xiaohong Wang, Xueqing Xia, Arun Satelli, Shulin Li

**Affiliations:** Department of Pediatrics–Research, The University of Texas MD Anderson Cancer Center, Houston, TX 77030 USA; Department of Experimental Radiation Oncology, The University of Texas MD Anderson Cancer Center, Houston, TX 77030 USA

**Keywords:** Immune checkpoint PD-L1, Bone marrow derived cells, Tumor microenvironment, CD11b, Chemotherapy resistance

## Abstract

**Background:**

Expression of programmed cell death ligand 1 (PD-L1) is an important process by which tumor cells suppress antitumor immunity in the tumor microenvironment. Bone marrow (BM)–derived immune cells are an important component of the tumor microenvironment. However, the link between PD-L1 induction on tumor cells and communication with BM cells is unknown.

**Results:**

This study demonstrates that BM cells have a direct effect in inducing PD-L1 expression on tumor cells, which contributes to the tumor cells’ drug resistance. This novel discovery was revealed using a co-incubation system with BM cells and tumor cells. BM cells from wild-type C57BL6 mice and the immune-deficient mouse strains B-cell^−/−^, CD28^−/−^, perforin^−/−^, and Rag2^−/−^ but not CD11b^−/−^ dramatically increased the expression of tumor cell surface PD-L1. This PD-L1 induction was dependent on CD11b-positive BM cells through direct contact with tumor cells. Furthermore, p38 signaling was activated in tumor cells after co-incubation with BM cells, whereas the expression of PD-L1 was remarkably decreased after co-culture of cells treated with a p38 inhibitor. The increase in PD-L1 induced by BM cell co-culture protected tumor cells from drug-induced apoptosis.

**Conclusions:**

PD-L1 expression is increased on tumor cells by direct contact with BM-derived CD11b-positive cells through the p38 signaling pathway. PD-L1 may play an important role in drug resistance, which often causes failure of the antitumor response.

**Electronic supplementary material:**

The online version of this article (doi:10.1186/s12964-015-0093-y) contains supplementary material, which is available to authorized users.

## Background

The tumor microenvironment is comprised of tumor cells and a variety of other cells including stem, stromal, and endothelial, and a wide range of immune cells [1]. Many of these nonmalignant cells are derived from bone marrow (BM) and are recruited by tumor cells to enhance their survival as well as primary tumor growth, invasion, and dissemination to distant organs [2]. Moreover, distinct BM-derived populations such as myeloid cell–derived suppressor cells [3-5], mesenchymal stem cells [6-8], and tumor-associated macrophages [9,10] have been shown not only to promote cancer cell metastasis but also to escape tumor immune surveillance through suppression of antitumor T-cell responses. Although several recent studies have found correlations between infiltration of particular immune cells into primary tumors and prognosis in cancer patients [9,11,12], the details of the mechanism by which BM-derived cells in the tumor microenvironment contribute to tumor progression and metastasis have not been fully established.

Programmed cell death 1 ligand 1 (PD-L1; also known as B7-H1 or CD274), a 40-kDa transmembrane protein belonging to the B7 family that negatively regulates T-cell signaling, is frequently upregulated in a number of different tumors, including melanoma, ovarian, lung, glioblastoma, breast, and pancreatic cancers [13-17]. PD-L1 expression on tumor cells may correlate with higher malignant grade of tumors and tumor growth [18]. Also, PD-L1 transmits immune-inhibitory signals through the programed cell death 1 (PD-1) receptor on T cells, which allows tumors to escape from immune surveillance [19].

Several studies have reported the mechanisms of regulation of PD-L1 on tumor cells. The release of inflammatory cytokines such as interferon-gamma (IFN-γ) activates the signal transducer and activator of transcription 3 (STAT3) pathway and subsequently upregulates PD-L1 expression on lymphoma and lung cancer cells [20]. It has been also reported that the MyD88/mitogen-activated protein kinase (MAPK) kinase/extracellular signal–regulated kinase (MyD88/MEK/ERK) pathway upregulates PD-L1 transcription, which is initiated by both IFN-γ and Toll-like receptor ligands, and activates nuclear factor–kappa B for PD-L1 transcription [21,22]. Additionally, the loss of phosphatase and tensin homolog (PTEN) activates the phosphatidylinositide 3-kinase/Akt (PI3K/Akt) and mammalian target of rapamycin (mTOR) pathways, which leads to upregulation of PD-L1 on glioma and breast cancer cells [23,24]. Recently, it has been shown that tumor cell surface PD-L1 expression is upregulated by activation of CD8^+^ T cells in the melanoma tumor microenvironment [25] and that microRNA miR-513 repressed the translation of PD-L1, whereas IFN-γ treatment decreased miR-513 expression and induced PD-L1 translation [26]. Thus, regulation of PD-L1 appears to result from complex interactions between environmental stimuli, intracellular signaling pathways, and both transcriptional and translational control mechanisms. However, little is known about the influence of tumor microenvironment on regulation of tumor cell surface PD-L1 expression.

In this study, we hypothesized that BM-derived cells in the tumor microenvironment may interact with tumor cells and induce tumor cell surface PD-L1 expression through cell-cell communication. We further hypothesized that the increased PD-L1 expression may protect tumor cells from chemotherapeutic drug treatment *via* increasing drug resistance of tumor cells. These results showed that PD-L1 expression on tumor cells was dramatically induced by direct interaction between BM cells and tumor cells. Notably, CD11b expression on BM cells was critical for PD-L1 expression on tumor cells. We also investigated the signaling mechanism leading to PD-L1 upregulation and demonstrated that the p38 pathway was involved. Together, these results reveal a previously undisclosed role for BM cells in inducing tumor cell surface PD-L1 expression and implicate the CD11b-positive BM cell population in this induction.

## Results

### Bone marrow cells induce PD-L1 expression on the tumor cell surface

**Figure 1:**
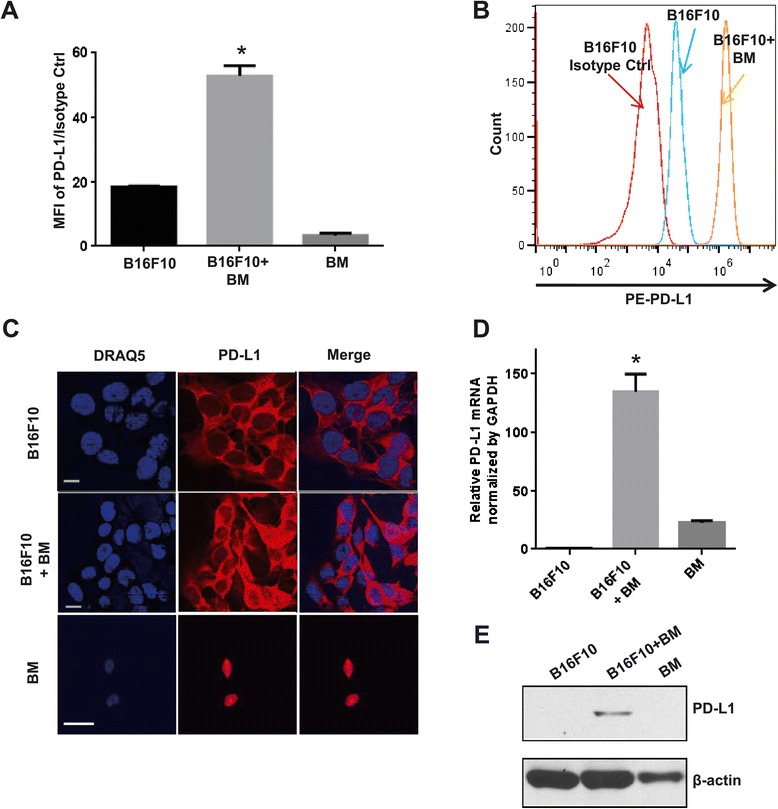
**Bone marrow cells induce PD-L1 expression on tumor cells. (A)** Tumor cell surface PD-L1 expression after co-culture with BM cells for 48 hours. Cells were stained with isotype control or PE-PD-L1 antibody. PD-L1 expression level was determined by flow cytometry. Data are presented as mean ± standard error (n = 3), *P <0.05 versus B16F10 alone. Student *t* test **(B)** Intracellular PD-L1 in B16F10 cells was detected by staining with isotype control or PE-PD-L1 antibody, and PD-L1 expression level was examined using flow cytometry. Results are representative of three independent experiments. **(C)** Immunostaining of PD-L1 (red) expression in B16F10 cells in monoculture or co-culture with BM cells. Nucleus (blue) was stained with DRAQ5. **(D)** Total RNA was isolated from B16F10 cells co-cultured with BM cells and then subjected to qRT-PCR to measure the level of PD-L1. As a control, mono-cultured B16F10 cells and BM cells were separately collected using Trizol and then followed total RNA isolation to measure the level of PD-L1. The levels of GAPDH were also determined and served as an internal control for standardization. Data are presented as mean ± standard error (n = 3), *P <0.05 versus control. **(E)** B16F10 cells were co-cultured with BM cells for 48 hours and subjected to lysis; cell lysates were subjected to immunoblotting to detect PD-L1. β-actin was used as a loading control. MFI = Median Fluorescence Intensity, BM = Bone Marrow.

PD-L1 expression on tumor cells limits T-cell activation, attenuates tumor immunosurveillance, and correlates with tumor growth and metastasis [18,19]. However, the effect of stromal cells in the tumor microenvironment on this PD-L1 expression has not been determined. This investigation focused, therefore, on the regulatory effect of the BM-derived stromal cells that often surround tumors on expression of PD-L1 on the tumor cell surface. The co-culturing of B16F10 mouse melanoma cells with freshly-isolated syngeneic BM cells from C57BL6 mice allowed for characterization of the contribution of BM cells in the tumor microenvironment. After 48 hours, tumor cell surface PD-L1 expression was dramatically induced by co-culture with these wild-type BM cells (Figure 1A). Importantly, BM-induced PD-L1 expression was detected in various other tumor cell lines, including osteosarcoma and breast cancer cells (Figure 1A and Additional file 1: Figure S1), which suggests BM-derived cell–induced PD-L1 expression on tumor cells is a general phenomenon and is not cell type specific. To investigate whether this induction of PD-L1 expression occurred throughout tumor cells or only on the cell surface, both intracellular and cell surface PD-L1 expression levels were determined in B16F10 cells by flow cytometry. The data show that total PD-L1 levels as well as surface expression were increased in the B16F10 melanoma cells (Figure 1B). Immunocytochemical staining and confocal microscopy of tumor cells confirmed the PD-L1 expression in B16F10 cells after co-culture with BM cells. PD-L1 expression was significantly greater in co-cultured B16F10 tumor cells than in the mono-cultured control B16F10 cells (Figure 1C). Taken together, these results suggest that BM cells induced PD-L1 expression within the tumor cells and then the induced PD-L1 translocated to the tumor cell surface. Western blot and qRT-PCR analysis showed that both PD-L1 protein and mRNA levels were increased in B16F10 cells after co-culture with BM cells (Figure 1D and E), further supporting the suggestion that BM cells upregulate PD-L1 gene expression.

### Direct contact between tumor and bone marrow cells is required for PD-L1 expression

**Figure 2:**
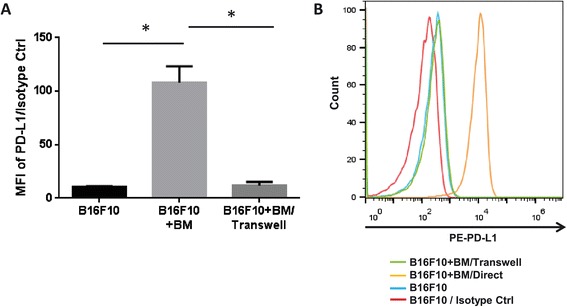
**Direct interaction between BM and tumor cells is required for PD-L1 expression.** Cell surface PD-L1 expression was detected on B16F10 cells in monoculture or co-culture with BM cells by staining with isotype control or PD-L1 antibody and flow cytometry. Data was represented with **(A)** Bar Graph, **(B)** Histogram. Data are presented as mean ± standard error (n = 3). *P <0.05 versus B16F10 alone, student *t* test. MFI = Median Fluorescence Intensity, BM = Bone Marrow.

To investigate whether induction of PD-L1 expression by BM cells is mediated by direct cell-to-cell contact or by soluble factors, we conducted an *in vitro* indirect co-culture experiment using the ThinCert™ transwell membrane. This membrane kept the two cell populations physically separated at all stages of the co-culture, while the pores of the membrane allowed the exchange of soluble factors between the two compartments. Unlike direct contact, indirect co-culture of B16F10 cells with BM cells did not induce PD-L1 expression on the tumor cell surface (Figure 2A and B). This result was further confirmed using DBT cells (Additional file 1: Figure S2). Taken together, tumor cells require direct contact to communicate with BM cells to induce surface PD-L1 expression.

### PD-L1 upregulation is dependent on CD11b-expressing BM cells in the tumor microenvironment

**Figure 3:**
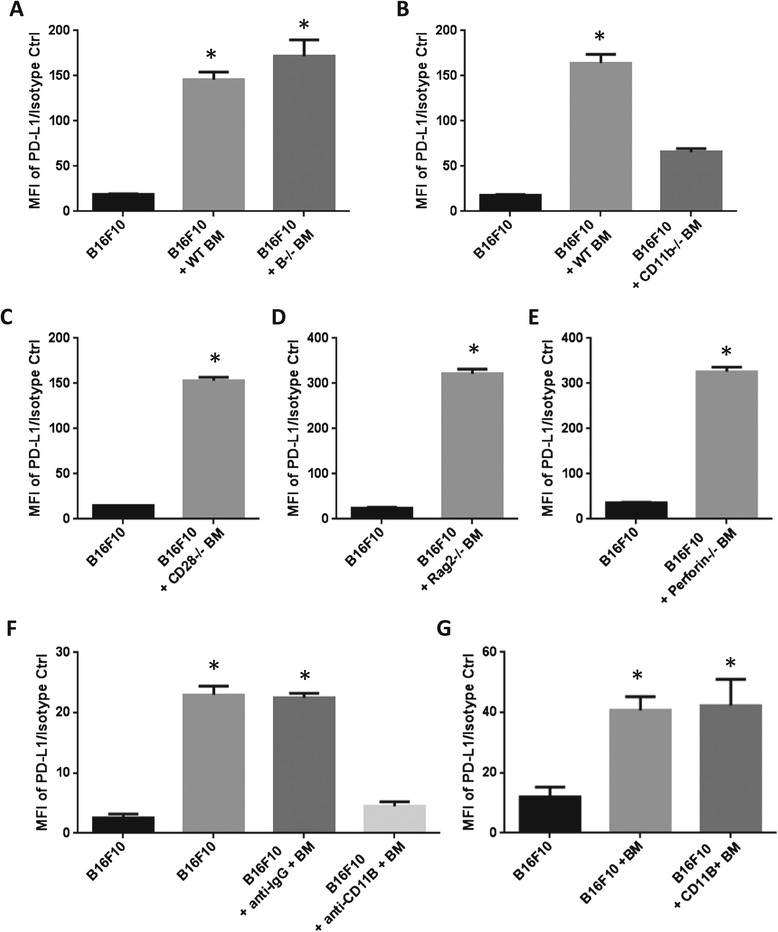
**CD11b-positive BM cells are critical for PD-L1 expression.** B16F10 tumor cell surface PD-L1 expression was determined after co-culture with BM cells isolated from knockout mice, including **(A)** B−/−, **(B)** CD11b−/−, **(C)** CD28−/−, **(D)** Rag2−/−, and **(E)** perforin−/− mice. **(F)** B16F10 cells were pre-incubated with anti-rat-IgG or CD11b-neutralizing antibody and co-cultured with BM cells. PD-L1 expression was determined by staining with isotype control or PE/Cy7-PD-L1 antibody and using flow cytometry. **(G)** CD11b-positive BM cells were separated from the mixed BM cells using PE-CD11b-coupled nanoparticles. B16F10 cells were co-cultured with CD11b-positive BM cells for 48 hours and then stained with isotype control or PE/Cy7-PD-L1 antibody. PD-L1 expression level was determined by flow cytometry. Data are presented as mean ± standard error (n = 3), *P <0.05 versus B16F10 alone, student *t* test. MFI = Median Fluorescence Intensity, BM = Bone Marrow.

To identify which BM subpopulation is responsible for PD-L1 expression on tumor cells, BM cells from different knockout mice deficient in CD28, perforin, B cells, Rag2, or CD11b were isolated and co-incubated with B16F10 cells. After 48 hours, tumor cell surface PD-L1 expression was examined using flow cytometry. All of the BM cells from different knockout mice retained the capacity to induce surface PD-L1 expression in B16F10 cells (Figure 3A, C, D and E) except those deficient in CD11b (Figure 3B). These data show that tumor cells may regulate PD-L1 expression on tumor cells via communication with CD11b-positive BM cells in the tumor microenvironment and that CD28-positive cells, natural killer cells, B cells, and T cells are not critical for induction of tumor cell surface PD-L1 expression. To confirm the role of CD11b in the communication between tumor and BM cells, CD11b-neutralizing antibodies were added during the co-culture with BM cells, leading to significantly lower PD-L1 expression on the tumor cells (Figure 3F). The importance of CD11b in PD-L1 induction on B16F10 cells was further confirmed by co-culture with CD11b-positive BM cells separated from the mixed BM cells, showing PD-L1 induction on the tumor cells similar to co-culturing with total BM cells (Figure 3G). Taken together, these results indicate that CD11b-expressing BM cells communicate with tumor cells in the tumor microenvironment to induce tumor cell surface PD-L1 expression.

### Bone marrow cells induce PD-L1 expression on tumor cells in a p38 pathway–dependent manner

**Figure 4:**
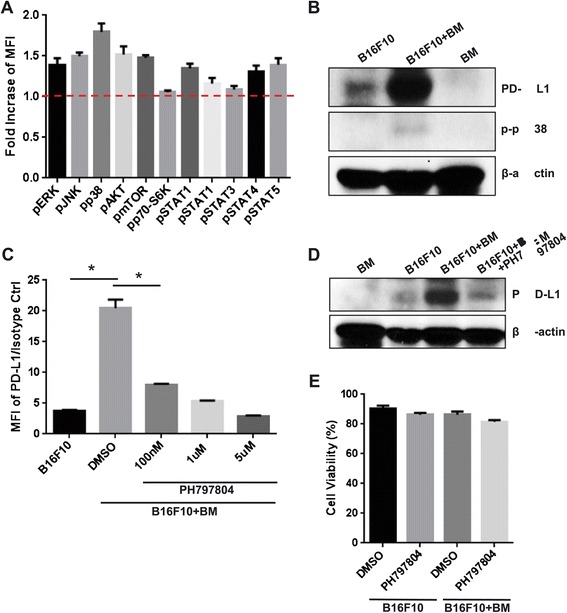
**Induction of PD-L1 by BM cells is dependent on the p38 signaling pathway. (A)** B16F10 cells co-cultured with BM cells were stained with pERK, pJNK, pp38, pAKT, pmTOR, pp70-S6K, pSTAT1, pSTAT3 pSTAT4, and pSTAT5 antibodies and analyzed using flow cytometry. Fold increase represents the MFI ratio between co-culture and monoculture (MFI of B16F10 in co-culture/MFI of B16F10 in monoculture). **(B)** B16F10 cells co-cultured with BM cells were subjected to lysis, and cell lysates were subjected to immunoblotting to detect PD-L1 and p-p38 levels. β-actin was used as a loading control. **(C)** PD-L1 expression was determined in B16F10 cells co-cultured with BM cells and p38 inhibitor PH797804 by staining with PD-L1 antibody and flow cytometry analysis. **(D)** B16F10 cells were treated with 1 μM PH797804 during co-culture with BM cells for 48 hours. Cells were subjected to lysis, and cell lysates were subjected to immunoblotting to detect PD-L1. β-actin was used as a loading control. **(E)** B16F10 cells were treated with 5 μM PH797804 during monoculture or co-culture with BM cells for 48 hours and then stained with annexin V and PI to determine cell viability by flow cytometry. Data are presented as mean ± standard error (n = 3). *P <0.05 versus B16F10 alone, student *t* test. MFI = Median Fluorescence Intensity, BM = Bone Marrow.

Next, we looked into the molecular pathways regulating induction of tumor cell surface PD-L1 expression by BM cells. Several signaling pathways, including the STAT3, MAPK, and PI3K pathways, have been shown to regulate PD-L1 expression on cancer cells [20-24]. Thus, the activation of signaling components, including ERK, JNK, p38, AKT, mTOR, p70-S6K, and STATs, was examined *via* intracellular staining and flow cytometry in B16F10 cells after 48 hours of co-culture with BM cells. Although the STAT3 signaling pathway has been reported to regulate PD-L1 expression in the NPM/ALK-carrying T cell lymphoma (ALK + TCL) cells [20], BM cell interaction did not activate STAT3 in B16F10 tumor cells. In the western blot data, both BM and the mixture of B16F10 tumor cells plus BM cells showed the activated STAT3 (pSTAT3), but it is not possible to determine whether the pSTAT3 expression is from the tumor cells or BM cells (Additional file 1: Figure S3). Flow cytometry analysis clearly showed that pSTAT3 activation in co-cultured B16F10 cells was not increased compared to monocultured B16F10 cells (Figure 4A). The activation of p38 in tumor cells was markedly increased compared to that of the others after BM cell co-culture (Figure 4A). The increased expression of p-p38 protein was confirmed by western blotting (Figure 4B). To test whether the p38 pathway is critical for tumor cell surface PD-L1 upregulation, we added a p38-specific inhibitor, PH797804, to the co-cultures and determined its effect on PD-L1 expression on B16F10 cells. This inhibitor abrogated BM cell–induced PD-L1 expression on B16F10 cells in a dose-dependent manner (Figure 4C). The suppression of PD-L1 expression by PH797804 was confirmed by western blotting (Figure 4D). Additionally, the B16F10 cells were still viable after the treatment of 5 μM PH797804 (Figure 4E). These data show that integral activation of p38 is required for the BM cell induction of PD-L1 expression on B16F10 tumor cells.

### Bone marrow cells protect tumor cells and increase drug resistance *via* upregulation of PD-L1 expression

**Figure 5:**
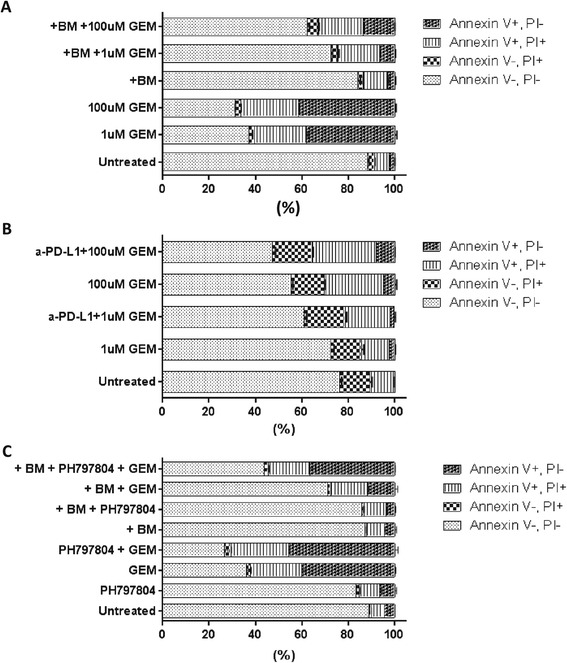
**BM cells increase drug resistance of tumor cells and protect them** ***via*** **upregulation of PD-L1. (A)** B16F10 cells were treated with gemcitabine during co-culture with BM cells for 48 hours and then stained with annexin V and PI to determine proportions of necrotic and apoptotic cells by flow cytometry. **(B)** B16F10 cells were pre-incubated with PD-L1–blocking antibody for 3 hours and then treated with gemcitabine during co-culture with BM cells. Proportions of necrotic and apoptotic cells were determined as in **(A)**. **(C)** B16F10 cells were treated with 5 μM PH797804 and 100 μM gemcitabine during co-culture with BM cells for 48 hours. Proportions of necrotic and apoptotic cells were determined as in **(A)**. Data are presented as mean ± standard error (n = 3). BM = Bone Marrow, GEM = Gemcitabine.

**Figure 6:**
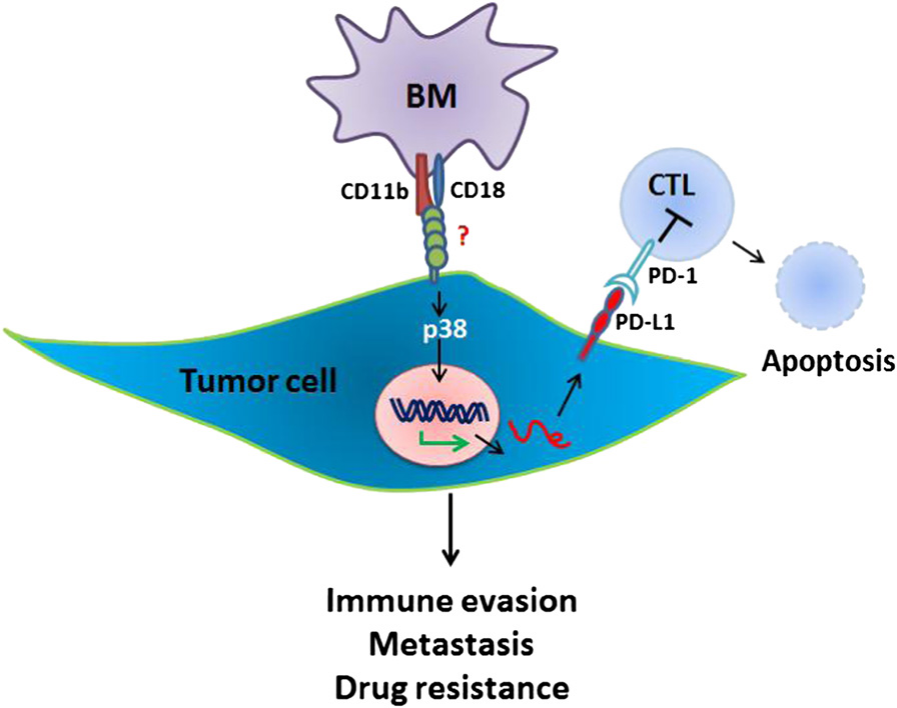
**Model of PD-L1 induction on tumor cells by CD11b-positive BM.** BM cells adhere to the surface of tumor cells through CD11b interaction with an unknown receptor on the tumor cell surface. This interaction activates the p38 signaling pathway and induces PD-L1 expression, both intracellular and on the tumor cell surface. Overexpressed PD-L1 may play a role in metastasis, immune evasion, and drug resistance.

A recent study showed that high PD-L1–expressing basal type breast cancer cell lines overexpress genes involved in chemoresistance compared to low PD-L1–expressing breast cancer cell lines [27]. So, it is possible that overexpression of PD-L1 on the tumor cell surface may be a mechanism whereby BM cells protect tumor cells from drug treatment. To test this hypothesis, B16F10 cells were treated with gemcitabine (1 μM or 100 μM) during monoculture or co-culture with BM cells. After 48 hours, the B16F10 cells were analyzed using Pacific blue–annexin V and PI staining to detect drug-induced apoptosis. The proportion of viable cells was higher in co-cultured gemcitabine-treated B16F10 cells than in mono-cultured treated B16F10 cells, whereas the proportions of apoptotic and necrotic cells in co-cultured gemcitabine-treated B16F10 cells were decreased (Figure 5A). This result indicates that BM-derived immune cells in the tumor microenvironment protect tumor cells from drug treatment. To investigate the role of tumor cell surface PD-L1 in the response to drug treatment, B16F10 cells were incubated with PD-L1 blocking antibodies (concentration at 2 μg/mL) prior to adding BM cells. B16F10 cells in which PD-L1 was blocked with PD-L1 antibodies were then co-cultured with BM cells and gemcitabine for 48 hours. Pacific blue–annexin V and PI staining showed that apoptosis and necrosis of B16F10 cells that had been inhibited by BM cells were increased by blocking PD-L1 after treatment with gemcitabine (Figure 5B), indicating the anti-apoptotic role of PD-L1 in tumor cells. Additionally, the role of p38 in the PD-L1 mediated drug resistance was further confirmed by the treatment of 5 μM PH797804 and 100 μM gemcitabine during co-culture with BM cells for 48 hrs. The proportion of viable cells was lower in co-cultured PH797804 and gemcitabine-treated B16F10 cells than in co-cultured gemcitabine treated B16F10 cells, whereas the proportions of apoptotic and necrotic cells in co-cultured PH797804 and gemcitabine-treated B16F10 cells were increased (Figure 5C). A possible acting model for this chemoresistance induction is depicted in Figure 6 and illustrates that suppression of antitumor T-cell response *via* the interaction between PD-1 on effector T cells and induced PD-L1 on tumor cells may lead to this resistance of drug treatment.

## Discussion

Tumor immune surveillance has been shown to be essential for tumor cell survival during tumor progression and metastasis. One of the major molecular regulators of tumor immune escape is PD-L1, which inhibits T cell–mediated immune attack through binding to its receptor PD-1 on tumor-specific T cells [28]. PD-L1 expression has been reported in several human malignancies and has been linked to poorer prognosis and increased resistance to anticancer therapies in many of these malignancies [29]. Although several mechanisms, such as PI3K and STAT3 signaling pathways *via* PTEN inhibition, of PD-L1 regulation on tumor cells have been reported [20,23,24], the cellular interactions between tumor cells and tumor stromal cells responsible for PD-L1 expression have remained unknown. Besides the pathways already reported for PD-L1 regulation, this report demonstrates for the first time that tumor cell surface expression of PD-L1 is induced through direct interaction with BM cells in the tumor microenvironment and that this effect is p38 dependent.

Over the last decade, the tumor microenvironment has been a topic of great interest, with the goal of understanding the contribution of tumor stromal components to regulation of tumor progression and metastasis [30]. BM-driven cells have been shown to have direct impact on tumor progression and metastasis by regulating angiogenesis, inflammation, and immune suppression. Moreover, increased BM recruitment by tumors has been associated with poor prognosis in clinical studies. Better prognoses have been observed when tumor sites are loaded with tumor-infiltrating lymphocytes (TILs) to aid the immune system in the clearance of tumor cells. However, PD-L1 positivity at tumor sites was also associated to TILs with poor prognoses, as tumor cells might exploit the PD-L1 expression to overcome antitumor immune responses mediated by TILs [29,31]. This observation strongly suggests that the oncogenic drivers for malignant transformation result in expression of the inducible ligand PD-L1 as a generalizable principle of the development of the malignant phenotype. Furthermore, the components of the BM-derived tumor microenvironment may be targeted and studied as a biomarker for cancer metastasis [32]. However, a role for BM cells in the regulation of PD-L1, a regulator of tumor immune surveillance, had not been addressed and was the subject of this study. This investigation of a putative role for BM cells in regulation of PD-L1 expression on tumor cells showed that cell surface PD-L1 expression was induced in different tumor cells by co-culture with BM cells. Furthermore, CD11b was critical for induction of PD-L1 expression by direct contact with BM. Additional investigations examined whether B16F10 cells expressed ICAM-1, a known receptor for CD11b, and whether engagement of the receptor was involved in PD-L1 expression on B16F10 cells during BM cell co-culture. ICAM-1 expression was detected on only ~25% of co-cultured B16F10 cells, whereas more than 90% of these cells expressed PD-L1 (Additional file 1: Figure S4). These results suggest that ICAM-1 was not the CD11b receptor associated with induction of PD-L1 expression on tumor cells during BM co-culture. The results presented in this study, therefore, point to the possibility of an unknown receptor on B16F10 tumor cells that bound BM CD11b to trigger PD-L1 expression.

The findings that the p38 MAPK pathway was activated by communication between BM and B16F10 tumor cells and mediated PD-L1 expression on the tumor cells are corroborated by the recent observation that p38 activation is important for poly I:C–induced PD-L1 expression in myeloid dendroid cells [33]. The expression of PD-L1 has been associated with MAPK signaling in other tumor types. The activation of Toll-like receptor 4 signaling by lipopolysaccharide stimulation induced PD-L1 expression in bladder cancer cells *via* activation of ERK and JNK [22]. Furthermore, PD-L1 expression was found to be regulated by MEK/ERK signaling in anaplastic large cell lymphoma and Hodgkin lymphoma, and the ERK and p38 MAPK signaling pathways were suggested to be involved in the regulation of PD-L1 in Hodgkin lymphoma cells [34]. Thus, this study underlines the important role of the p38 pathway in influencing PD-L1 expression and show, for the first time, this mode of action in the tumor microenvironment.

Finally, that BM cells protected B16F10 cells from gemcitabine treatment and this drug resistance was blocked by PD-L1 neutralization in B16F10 cells, indicating the significance of tumor cell surface PD-L1 in drug resistance of tumor cells. This result is in line with the anti-apoptotic role of cell surface PD-L1 in breast cancer cells [35]. That study showed that cell surface PD-L1 expression was downregulated by doxorubicin treatment and that this effect was accompanied by upregulation of PD-L1 in the nucleus, suggesting the role of cell surface PD-L1 in apoptosis. PD-L1 knockdown using siRNA led to an increase in spontaneous apoptosis as well as doxorubicin-induced apoptosis in these breast cancer cells [35]. Additionally, gemcitabine treatment in pancreas cancer patients showed no significant changes in proportions of T and B-cells including CD86 and CD80 APCs or CD4+, CD25+ T-cells [36]. Increased population of CD14+ monocytes and CD11C+ dendritic cells were also documented with gemcitabine treatment in patients with advanced pancreatic cancer [37]. PD-L1 is a negative co-stimulatory molecule that is expressed in many cancers, where it is believed to contribute to the escape of tumors from immune recognition through binding to its receptor, PD-1, on tumor-specific T cells [38]. Upregulation of PD-L1 has been implicated in the immune escape of several human malignancies and PD-L1–positive status is linked to tumor metastasis, poor survival, and increased risk of mortality in several human cancers [29]. Furthermore, blocking of PD-L1 or PD-1 with monoclonal antibodies has been demonstrated to trigger antitumor immune responses and enhance the effectiveness of anticancer immunotherapy [39]. Targeting this molecule may therefore lead to a major breakthrough in cancer treatment.

## Conclusions

This study demonstrates that BM cells expressing CD11b directly communicate with tumor cells and activate the p38 MAPK pathway (Figure 6). Tumor cell surface PD-L1 is overexpressed through this cell-cell contact in the tumor microenvironment, and this effect is dependent on the p38 signaling pathway. Overexpression of PD-L1 increases drug resistance and protects tumor cells from drug treatment. Further study of the CD11b–p38–PD-L1 signaling axis may lead to development of novel therapeutic targets for cancer. Further investigation of the unknown CD11b-interacting protein on tumor cells may be warranted.

## Materials and methods

### Cell culture and reagents

The cancer cell lines B16F10, DBT, 4 T1, LLC and LM8 were obtained from American Type Culture Collection (Rockville, MD, USA). B16F10, DBT, 4 T1, LLC and LM8 cells were cultured in Dulbecco modified Eagle medium (DMEM)/F12 (Sigma) supplemented with 10% fetal bovine serum and 10 U/ml penicillin and streptomycin (Life Technologies, Grand Island, NY) at 37°C in 5% CO_2_. Cells were detached using 1 mM EDTA in phosphate-buffered saline solution (PBS) and used for further experiment. Antibodies used for western blotting included PD-L1 (1:1000, R&D Systems, Minneapolis, MN), p-p38 and β-actin (1:1000, Santa Cruz Biotechnology, Dallas, TX). Antibodies for flow cytometry, including pp38, pERK, pJNK pAKT, pmTOR, pp70-S6K, pSTAT1, pSTAT3, pSTAT4 and pSTAT5, were purchased from Cell Signaling Technology.

### Animal ethics statement

The mice used in this study were maintained under National Institutes of Health guidelines and euthanized according to procedures approved by the Institutional Animal Care and Use Committee of The University of Texas MD Anderson Cancer Center.

### Isolation of bone marrow cells from mouse bones

Six-to eight-week-old C57BL6, B^−/−^, CD11b^−/−^, CD28^−/−^, Perforin^−/−^, and Rag2^−/−^ mice obtained from the National Cancer Institute or Jackson Laboratory (Bar Harbor, ME) were used for this study. The bones were isolated from the two rear legs of each mouse and washed with phosphate-buffered saline solution (PBS) and DMEM/F12 medium. BM cells were flushed from both ends of the bones into cell culture dishes by injecting DMEM/F12 medium via a 26-gauge needle and a 10-mL syringe. Collected cells were subjected to lysis by red blood cell lysis buffer for 4 minutes at room temperature. BM cell lysate suspensions were passed through a 40-μM strainer and washed twice with RPMI-1640 medium. The cells were resuspended in 4 mL of RPMI-1640 medium and combined for co-culture with tumor cells.

### *In vitro* bone marrow co-culture

Tumor cells were labeled with carboxyfluorescein diacetate succinimidyl ester (CFSE, Biolegend, San Diego, CA) at a final concentration of 5 μM for 10 minutes at 37°C in darkness. After two washes with RPMI-1640 medium, the CFSE-labeled tumor cells were combined with freshly isolated BM cells in cell culture plates at the ratio 1:10. After 48 hours of co-incubation, BM cells were gently removed from the cell culture suspension and adherent tumor cells were detached and collected for further study. To investigate the manner of cell-cell communication, BM cells were incubated on ThinCertTM cell culture inserts (Greiner Bio-One) with translucent membranes and 0.4 μm pores, and B16F10 cells were cultured on the underside of the membrane for 48 hrs. To test the role of the CD11b subpopulation of BM cells, 2 μg/mL of anti-CD11b neutralization antibody (BS Pharmingen, San Jose, CA) was added to co-cultures. CD11b-positive cells were separated using EasySepTM Mouse CD11b positive selection kit (Stemcell technologies, Vancouver, Canada) and co-cultured with B16F10 cells. To examine the activity of the p38 pathway, p38 inhibitor PH797804 (Selleckchem, Houston, TX) was added into the culture medium at a final concentration of 1 μM.

### Flow cytometry

CFSE-stained tumor cells harvested from co-cultures were stained for PD-L1 expression. Briefly, tumor cells were blocked for 10 minutes at room temperature with FcR blocker in a 1:1000 dilution and then incubated with anti-PD-L1 antibody (phycoerythrin [PE]-conjugated anti-mouse PD-L1, Biolegend) in a 1:50 dilution in PBS + 2% serum for 15 minutes in the dark at room temperature. To determine the activation of signaling, tumor cells were fixed with 2% paraformaldehyde and permeabilized with 100% methanol, followed by intracellular staining with primary antibodies for p-p38, pERK, pJNK, pAKT, pmTOR, pp70-S6K, pSTAT1, pSTAT3, pSTAT4, and pSTAT5 and PE-conjugated rabbit IgG secondary antibody (Santa Cruz Biotechnology). Cells were analyzed on an Attune flow cytometer (Life Technologies, Grand Island, NY) and the results evaluated using FlowJo 10.0 software (Tree Star, Inc., Ashland, OR). CFSE-positive tumor cells were gated for further analyses of the expression of PD-L1 and intracellular cell signaling molecules. Gene expression was evaluated by Median Fluorescence Intensity (MFI).

### Western Blot

Same amount of total proteins isolated using Radioimmunoprecipitation assay (RIPA) buffer were loaded onto 12% sodium dodecyl sulfate-polyacrylamide gel (SDS-PAGE) and transferred to nitrocellulose membranes using the iBlot gel transfer device (Invitrogen, Grand Island, NY). The membranes were blotted with anti-PD-L1- or aniti-p-p38-primary antibody and HRP-conjugated secondary antibody (Santa Cruz Biotechnology, Dallas, TX) to detect the protein of interest.

### Quantitative Reverse transcription polymerase chain reaction (qRT-PCR)

Total RNA was extracted from cells using Trizol (Invitrogen) and quantitative reverse transcriptase PCR(qRT-PCR) was performed as previously described [40] to measure the levels of PD-L1. The levels of GAPDH mRNA were also measured and used as the internal normalization control. The forward and reverse primer sequences for the mouse PD-L1 and GAPDH are 5′-ACAGCCAGGGCAAAACCA-3′ (forward), 5′-GGATGTGTTGCAGGCAGTTCT-3′ (reverse) for PD-L1, and 5′-CCAGCCTCGTCCCG TAGAC-3′(forward), 5′-CGCCCAATACGGCCAAA-3’ (reverse) for GAPDH.

### Immunofluorescence imaging

For immunofluorescence imaging, the cells were cultured in chamber slides (Fisher scientific) and performed as previously described [41] to detect PD-L1 expression. After fixation using 4% paraformaldehyde (Fisher scientific), cells were washed in PBS (pH 7.4) and blocked in blocking buffer (1% FBS in PBS with 0.01% NP40) for an hour. Later, cells were incubated with PD-L1 antibody (1:1000 in blocking buffer) overnight in cold room. Cells were then rinsed in PBS and stained with Alexa Fluor-555 secondary antibodies (Invitrogen) (1:250) for PD-L1 staining (species: rabbit). For nuclei staining, DRAQ5 (CellSignaling) (1:1000) was incorporated along with secondary antibody for 60 min. The cells were then washed with PBS (pH 7.4) three times for 15 min each and mounted in Slow fade antifade (Invitrogen). For confocal analysis, images were acquired in 8 bits with the Zeiss LSM 510 confocal microscope using LSM 5 3.2 image capture and analysis software (Zeiss). A 63× water-immersion objective lens (NA, 1.0) was used with digital zoom for image capture. All images were acquired by the same operator using the same intensity and photo detector gain in order to allow quantitative comparisons of relative levels of immunoreactivity between different samples.

### Annexin V and propidium iodide staining

B16F10 cells were co-cultured with fresh BM cells with or without 1 μM gemcitabine for 48 hours. To test the role of PD-L1 in cell response to the drug, B16F10 cells were pre-incubated with 2 μg/mL of PD-L1 blocking antibody (eBioscience, San Diego, CA) for 2 hours before co-culture with freshly isolated BM cells and treatment with gemcitabine (1 μM). To test cell viability, B16F10 cells were treated with 5 μM PH797804 during co-culture. To examine the role of p38 in the PD-L1 mediated drug resistance, B16F10 cells were treated with 5 μM PH797804 and 100 μM gemcitabine. After 48 hours of co-culture, single-cell suspensions were prepared with cold PBS buffer. After two washes, cells (1 × 10^6^ cells/mL) were resuspended in 500 μL of annexin V binding buffer (Biolegend). Aliquots (100 μL) of the cell suspension were incubated with 5 μL of Pacific blue–conjugated annexin V (Biolegend) and 5 μL of propidium iodide (PI) solution (Biotium, Hayward, CA) for 15 minutes at room temperature in darkness. After staining, 400 μL of annexin binding buffer was added to the cells, which were immediately analyzed by flow cytometry.

### Statistical analysis

Results are expressed as mean ± standard deviation. Data were analyzed with GraphPad software (GraphPad Software, Inc., La Jolla, CA) using an unpaired two-tailed Student *t*-test to detect the significance of differences between groups. P <0.05 was considered statistically significant.

## Additional file

Additional file 1:
**Bone Marrow cells induce PD-L1 expression on tumor cells.** (A) LM8, (B) 4 T1, and (C) LLC tumor cell surface PD-L1 expression after co-culture with BM cells for 48 hrs. Cells were stained with isotype control or PE-PD-L1 antibody. PD-L1 expression level was determined using flow cytometry. Data are presented as mean ± standard error (n = 3). *, P < 0.05 versus B16F10 alone, student t test. S2. Direct interaction between BM and tumor cells is required for PD-L1 expression. DBT brain tumor cells were co-cultured with BM cells together or separately using transwell membrane. Cells were stained with isotype control or PE-PD-L1 antibodies, followed flow cytometry analysis. (A) Bar graph, Data are presented as mean ± standard error (n = 3). (B) Histogram *, P < 0.05 versus B16F10 alone, student t test. S3. pStat3 was not activated by BM co-culture in B16F10 cells. B16F10 cells co-cultured with BM cells were subjected to lysis, and cell lysates were subjected to immunoblotting to detect pStat3 levels. β-actin was used as a loading control. S4. PD-L1 induction is not correlated with ICMA-1 on tumor cells. B16F10 tumor cells were co-cultured with BM cells for 2 days. Cells were stained with isotype control, PE/Cy7-PD-L1 or PE-ICAM-1 antibodies, followed flow cytometry analysis. Results are representative of three independent experiments.

## Abbreviations

BM: Bone marrow
PD-L1: Programmed cell death 1 ligand 1
PD-1: Programmed cell death 1 receptor
IFN-γ: Interferon gamma
STAT: Signal transducer and activator of transcription
MAPK: Mitogen-activated protein kinase
ERK: Extracellular signal–regulated kinase
PTEN: Phosphatase and tensin homolog
PI3K: Phosphatidylinositide 3-kinase
mTOR: Mammalian target of rapamycin
DMEM: Dulbecco modified Eagle medium
PBS: Phosphate-buffered saline solution
CFSE: Carboxyfluorescein diacetate succinimidyl ester
PE: Phycoerythrin
PI: Propidium iodide
qRT-PCR: Quantitative reverse-transcriptase polymerase chain reaction
TIL: Tumor-infiltrating lymphocyte
PE/Cy7: Phycoerythrin-cyanine 7
