# Supplementary material for: Immune checkpoint regulator PD-L1 expression on tumor cells by contacting CD11b positive bone marrow derived stromal cells
Source: Cell Commun Signal. 2015 Feb 27;13:14. doi: 10.1186/s12964-015-0093-y (PMC4353689; doi:10.1186/s12964-015-0093-y)
Supplement: Additional file 1: — Bone Marrow cells induce PD-L1 expression on tumor cells. (A) LM8, (B) 4 T1, and (C) LLC tumor cell surface PD-L1 expression after co-culture with BM cells for 48 hrs. Cells were stained with isotype control or PE-PD-L1 antibody. PD-L1 expression level was determined using flow cytometry. Data are presented as mean ± standard error (n = 3). *, P < 0.05 versus B16F10 alone, student t test. S2. Direct interaction between BM and tumor cells is required for PD-L1 expression. DBT brain tumor cells were co-cultured with BM cells together or separately using transwell membrane. Cells were stained with isotype control or PE-PD-L1 antibodies, followed flow cytometry analysis. (A) Bar graph, Data are presented as mean ± standard error (n = 3). (B) Histogram *, P < 0.05 versus B16F10 alone, student t test. S3. pStat3 was not activated by BM co-culture in B16F10 cells. B16F10 cells co-cultured with BM cells were subjected to lysis, and cell lysates were subjected to immunoblotting to detect pStat3 levels. β-actin was used as a loading control. S4. PD-L1 induction is not correlated with ICMA-1 on tumor cells. B16F10 tumor cells were co-cultured with BM cells for 2 days. Cells were stained with isotype control, PE/Cy7-PD-L1 or PE-ICAM-1 antibodies, followed flow cytometry analysis. Results are representative of three independent experiments. [file 12964_2015_93_MOESM1_ESM.pdf]

# Immune checkpoint regulator PD-L1 expression on tumor cells by contacting CD11b positive bone marrow derived stromal cells

**Hyangsoon Noh [1*], Jiemiao Hu[1*], Xiaohong Wang[2*], Xueqing Xia [1], Arun Satelli[1], and Shulin Li[1#]**

**Supplemental S1**

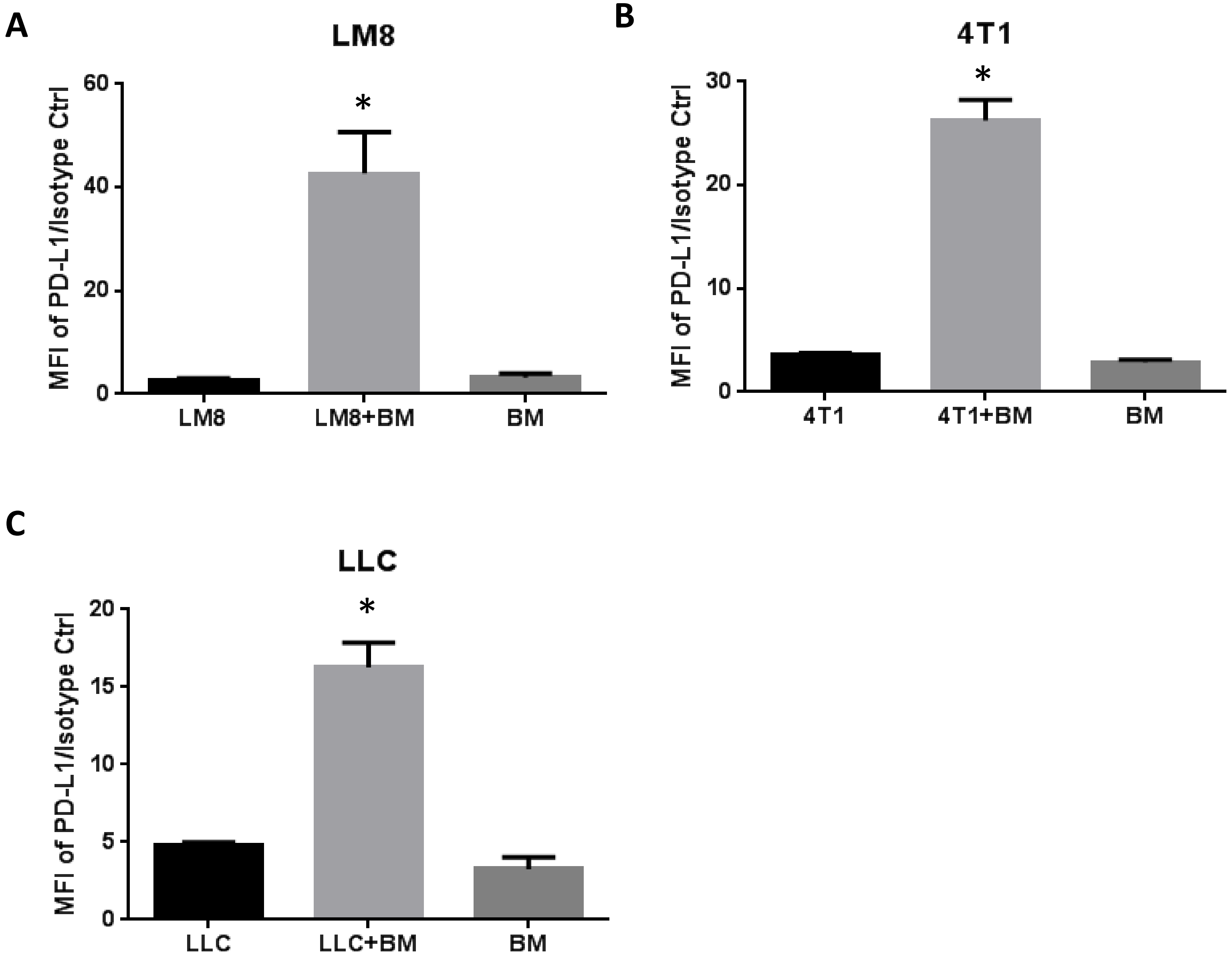


**S1. Bone Marrow cells induce PD-L1 expression on tumor cells.** (A) LM8, (B) 4T1, and (C) LLC tumor cell surface PD-L1 expression after co-culture with BM cells for 48hrs. Cells were stained with isotype control or PE-PD-L1 antibody. PD-L1 expression level was determined using flow cytometry. Data are presented as mean ± standard error (n=3). *, $P < 0.05$ versus B16F10 alone, student *t* test.

## Supplemental S2

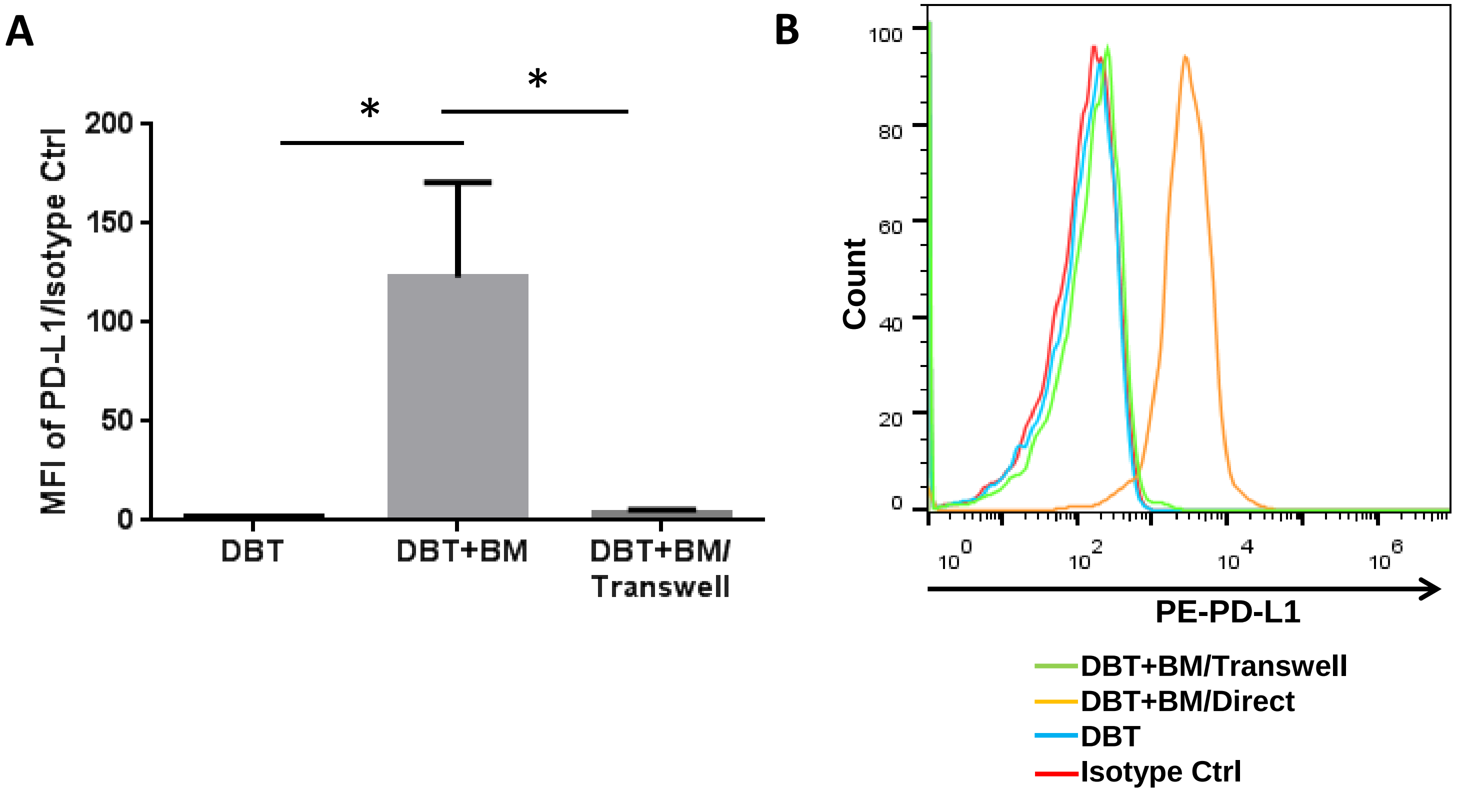


**S2. Direct interaction between BM and tumor cells is required for PD-L1 expression.** DBT brain tumor cells were co-cultured with BM cells together or separately using transwell membrane. Cells were stained with isotype control or PE-PD-L1 antibodies, followed flow cytometry analysis. (A) Bar graph, Data are presented as mean ± standard error (n=3). (B) Histogram *, P < 0.05 versus B16F10 alone, student *t* test.

**Supplemental S3**

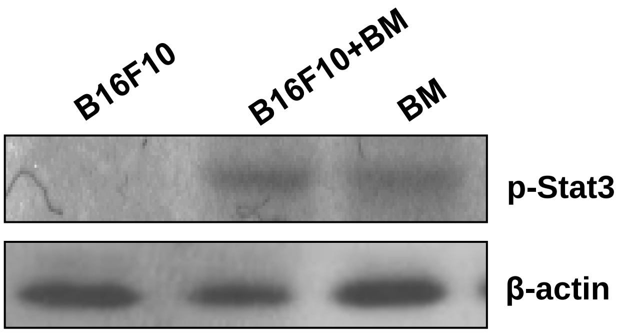


**S3. pStat3 was not activated by BM co-culture in B16F10 cells.** B16F10 cells co-cultured with BM cells were subjected to lysis, and cell lysates were subjected to immunoblotting to detect pStat3 levels. β-actin was used as a loading control.

**Supplemental S4**

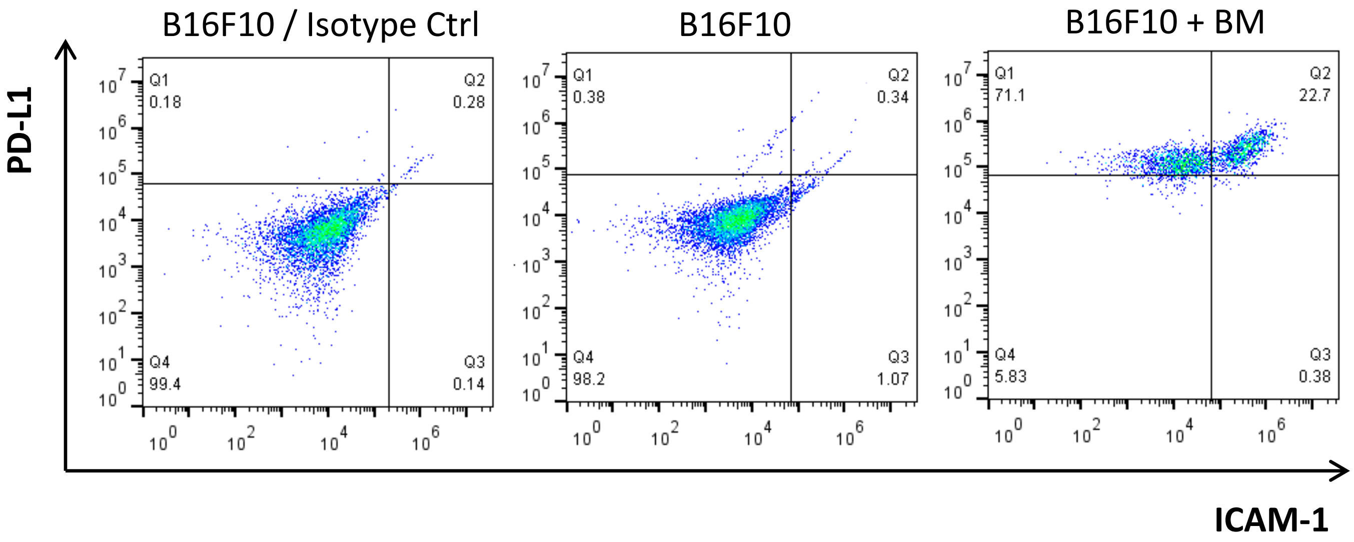


**S4. PD-L1 induction is not correlated with ICMA-1 on tumor cells.** B16F10 tumor cells were co-cultured with BM cells for 2 days. Cells were stained with isotype control, PE/Cy7-PD-L1 or PE-ICAM-1 antibodies, followed flow cytometry analysis. Results are representative of three independent experiments.
